# Premature delivery impacts the concentration of plasminogen activators and a plasminogen activator inhibitor and the plasmin activity in human milk

**DOI:** 10.3389/fped.2022.917179

**Published:** 2022-08-09

**Authors:** Veronique Demers-Mathieu, Mark A. Underwood, David C. Dallas

**Affiliations:** ^1^Nutrition Program, School of Biological and Population Health Sciences, College of Public Health and Human Sciences, Oregon State University, Corvallis, OR, United States; ^2^Department of Pediatrics, University of California, Davis, Sacramento, CA, United States

**Keywords:** premature neonates, breastfeeding, lactation, enzymes, digestive system, proteases, newborns, immaturity

## Abstract

**Background and aims:**

Plasmin in human milk partially hydrolyzes milk proteins within the mammary gland and may enhance the hydrolysis of milk proteins within the infant’s stomach. This study examined the effects of extremely preterm (EP)-, very preterm (VP)-, and term-delivery on plasmin activity and the concentrations of plasminogen activators [urokinase-type plasminogen activator (uPA) and tissue-type plasminogen activator (tPA)], plasminogen activator inhibitor type 1 (PAI-1) and the complexes of PAI-1/uPA and PAI-1/tPA in human milk.

**Materials and methods:**

Human milk samples were collected from mothers who delivered extremely preterm infants [24–27 weeks gestational age (GA), *n* = 20], very preterm infants (28–32 weeks GA, *n* = 12), and term infants (38–39 weeks GA, *n* = 8) during 2–72 days postnatally. Plasmin activity was determined using fluorometric substrate assay, whereas concentrations of uPA, tPA, PAI-1, the PAI-1/uPA complex and the PAI-1/tPA complex were quantified by ELISA.

**Results:**

Plasmin activity, uPA and tPA were detected in all human milk samples, PAI-1 and the PAI-1/uPA complex were present in 42.5 and 32.5% of milk samples, respectively, and the PAI-1/tPA complex was not detected. Plasmin activity was correlated negatively with postnatal age and postmenstrual age (PMA) in the VP group and positively with postnatal age in the term group. uPA and tPA concentrations decreased with increasing postnatal age in both EP and VP groups but did not correlate in the term group. uPA concentration was correlated positively with GA in the VP group and tended to be elevated with increasing GA in the combined three groups. In contrast, tPA concentrations were correlated negatively with GA and PMA in the combined three groups (*P* < 0.008) and with PMA in the EP and VP groups. PAI-1 concentration tended to be correlated positively with postnatal age in the combined three groups. No correlation was detected with the PAI-1/uPA complex.

**Conclusion:**

Premature delivery impacted the plasmin activity and the concentrations of uPA, tPA, and PAI-1 in human milk. Whether these changes in milk plasminogen activators and inhibitors have a role in balancing the proteolytic digestion of premature infants remains to be investigated.

## Graphical Abstract

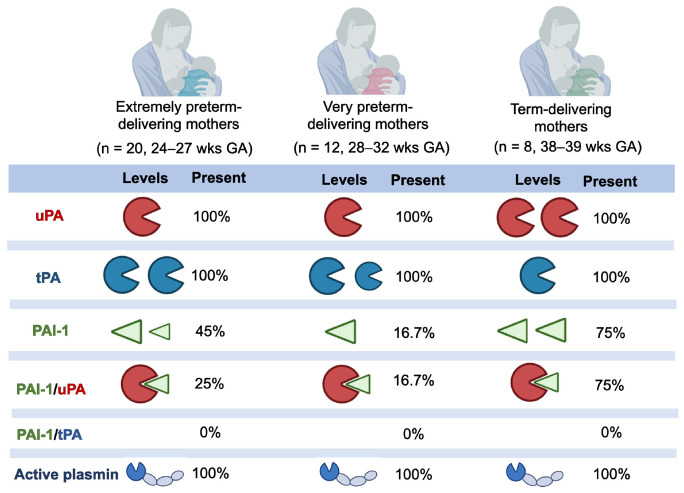

## Introduction

Human milk contains zymogens, active proteases, protease activators, protease activator inhibitors, and protease inhibitors that modulate the partial hydrolysis of milk proteins within the mammary gland (1) and may modulate the infant’s digestive capacity (2). Human milk contains several complex and interconnected proteolytic systems, and the balance of these systems controls the overall protease activity within the milk (3, 4). Evidence suggests that these proteases are initially active, releasing peptides from milk proteins even within the mammary gland prior to expression (1). We also found that carboxypeptidase B2, kallikrein, plasmin, elastase, thrombin, and cytosol aminopeptidases were active in human milk and that procathepsin D was present but not active. Among these, only plasmin and cathepsin D from human milk actively hydrolyzed proteins at gastric pH (2). These observations imply that plasmin can continue hydrolyzing milk proteins within the infant stomach.

Some research has examined whether the proteolytic system within human milk differs across gestational age of the infant at delivery. Our previous peptidomics-based analysis indicated that milk protease activity and peptide release were higher in the milk of preterm-delivering mothers than term-delivering mothers (5). Moreover, among mothers delivering prematurely, we found that plasmin, kallikrein, and thrombin activity was higher in milk from mothers who delivered at early gestational age (24–26 weeks GA) than those who delivered at late gestational age (GA) (27–32 weeks GA), especially at an early postnatal age (6). However, direct measurement of overall milk protease activity revealed no differences between human milk samples from the term-and preterm-delivering mothers (7).

The role of human milk proteases in overall protein digestion within the preterm and term infant is unclear. Our previous study demonstrated that preterm infants had a lower gastric digestive capacity (proteolysis and protease activity) than term infants (7). Variation in the proteolytic system of human milk with gestational age at delivery may contribute to the observed digestive capacity difference between preterm and term infants.

**FIGURE 1:**
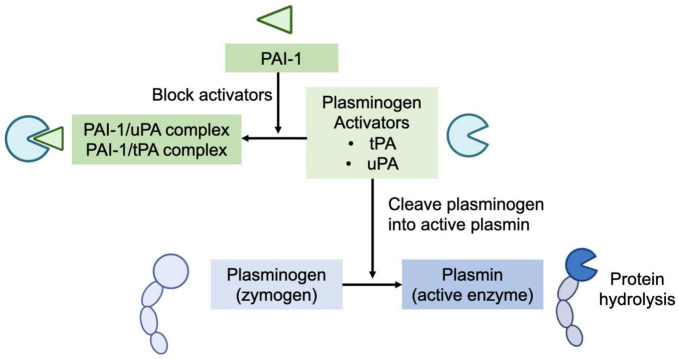
Human milk’s plasmin system is composed of plasminogen (zymogen), plasmin (the active enzyme), urokinase-type plasminogen activator (uPA), and tissue-type plasminogen activator (tPA) (plasminogen activators), and human plasminogen activator inhibitor type 1 (PAI-1) (plasminogen activator inhibitor). Plasminogen (the inactive zymogen) must be cleaved by uPA or tPA to become the active plasmin. The PAI-1 in human milk can block the activators’ (both tPA and uPA) conversion of plasminogen to active plasmin.

Human milk’s plasmin system is composed of plasminogen (zymogen), plasmin (the active enzyme), urokinase-type plasminogen activator (uPA), and tissue-type plasminogen activator (tPA) (plasminogen activators), and human plasminogen activator inhibitor type 1 (PAI-1) (plasminogen activator inhibitor) (Figure 1) and several potential direct plasmin inhibitors, including α-1-antitrypsin and α-2-antiplasmin (4). Plasminogen (the inactive zymogen) must be cleaved at a specific peptide bond (Arg 560) by uPA or tPA to become the active serine protease plasmin (3, 8). The PAI-1 in human milk can block the activators’ (both tPA and uPA) conversion of plasminogen to active plasmin (9).

Our previous enzyme-substrate assay analysis revealed that plasmin is active in human milk and remains active in the infant’s stomach (2). Yet, the concentrations of key components in the plasmin system in human milk remain unexplored, including the concentrations of uPA and PAI-1. Moreover, no research has yet evaluated the impact of gestational age of the infant at delivery on the overall plasmin system in human milk. Specifically, whether the milks of mothers who delivered extremely preterm (EP), very preterm (VP) or at term differ in plasmin activity and in concentrations of tPA, uPA, PAI-1, the PAI-1/uPA complex and the PAI-1/tPA complex remained unknown. Therefore, our aim in this study was to quantify the plasmin system components in human milk and determine whether premature delivery affects these components.

## Materials and methods

### Study design and participants

The Institutional Review Boards of the University of California, Davis (UC Davis) (289041-5) and Oregon State University (OSU) (7080) approved this study. We enrolled lactating women in the UC Davis Children’s Hospital Neonatal Intensive Care Unit in Sacramento, California. Informed consent was obtained from all mothers participating in the study. We collected human milk samples from 20 mothers who delivered EP infants (24–27 weeks GA), 12 mothers who delivered VP infants (28–32 weeks GA) and 8 mothers who delivered term infants (38–39 weeks GA) during 2–72 days postnatally (Table 1). The inclusion criteria were lactating women with an infant admitted to the neonatal intensive care unit (NICU).

**TABLE 1:** Demographic description of lactating women who donated human milk samples.

Demographics	Extremely preterm (*n* = 20)	Very preterm (*n* = 12)	Term (*n* = 8)	*P*-values
Postnatal age, days^a,b^	29 ± 17 (2–72)	23 ± 13 (5–45)	23 ± 10 (14–42)	0.42
Gestational age (GA), weeks^a^	25.0 ± 0.6 (24.1–27.0)	30 ± 2 (28–32)	38.9 ± 0.4 (38.0–39.4)	< 0.0001
PMA, days^a,c^	204 ± 18 (171–254)	232 ± 14 (210–254)	296 ± 9 (287–315)	< 0.0001
Birth weight, kg^a^	0.72 ± 0.08 (0.62–0.90)	1.3 ± 0.4 (1.0–2.2)	3.5 ± 0.3 (3.0–3.8)	< 0.0001
Head circumference, cm^a^	22.4 ± 0.8 (21.5–24.0)	27 ± 2 (25–32)	33.7 ± 0.4 (33–34)	< 0.0001
Infant biological sex, *n*	10 males: 11 females	5 males: 7 females	5 males: 3 females	0.66
Maternal age, years^a^	29 ± 6 (17–37)	31 ± 4 (26–40)	26 ± 8 (17–42)	0.11

^a^Data are presented as mean ± SD (min and max).

^b^Postnatal age at the time of milk collection.

^c^Postmenstrual age (PMA) represents the GA at birth plus the postnatal age.

### Human milk collection

One milk sample was collected from each mother on-site or at home with clean electric breast pumps into sterile plastic containers and stored immediately at −20°C. The breast was cleaned with water on a washcloth (no soap or alcohol) before pumping. Samples were transported to Oregon State University on dry ice and thawed on ice, and five 0.5-mL aliquots were placed into vials and stored at −80°C. Frozen samples were rapidly thawed at 37°C and centrifuged at 1,301 × *g* for 20 min at 4°C. The relatively low-speed centrifugation allowed lipid removal without precipitation and loss of casein micelles. The fat layer was removed, and defatted milk was collected for the measurements.

### Concentrations of plasminogen activators and the plasminogen activator inhibitor

The concentrations of human urokinase-type plasminogen activator (uPA), human tissue-type plasminogen activator (tPA), human plasminogen activator inhibitor type 1 (PAI-1), human PAI-1/uPA complex and PAI-1/tPA complex were determined using ELISA kits (IHUPAKT, IHTPAKT, IHPAIKT, IHPAIUPAKT-COM, and HPAITPAKT-COM, Innovative Research, Inc.) according to the protocols described by the manufacturer.

### Plasmin activity

The plasmin activity in the defatted milk samples was determined using a plasmin activity assay kit (K381-100, BioVision) according to the methods described by the manufacturer. Briefly, the standard curve was prepared by diluting 10 ng/μL plasmin standard in serial dilutions to prepare 50, 100, 150, 200, and 250 ng/well of plasmin standards. Milk samples (undiluted, 50 μL/well) and standards (50 μL/well) were added to the microplate. The substrate-fluorophore 7-amino-4-methylcoumarin (AMC) complex solution (50 μL) was mixed into each well. Fluorescence was measured (Ex/Em = 360/450 nm) on two replicates of blanks, standards and samples at 0 min (T0), 30 min (T30) and 60 min (T60) at 37°C with a microplate reader (Spectramax M2; Molecular Devices) with SoftMax Pro 4.8 (Molecular Devices). Plasmin activity (based on the release of AMC from the peptide substrate-AMC complex by plasmin) in samples was interpolated from the plasmin standard curve. T0 was subtracted from all readings (T60 and T30) and the final plasmin activity was reported as T60–T30 (μg of plasmin/mL/min).

### Statistical analysis

As the data failed the normality of distribution, Spearman’s rank correlation coefficient test was performed to evaluate the measurements (plasmin activity and concentrations of uPA, tPA, PAI-1, and the PAI-1/uPA complex) across postnatal age at sampling, GA at birth, postmenstrual age (PMA) at sampling, and maternal age. All correlations were bivariate, and no test controlling for any demographic or other confounding factors was performed due to the small sample size. The Mann-Whitney *U* test was used to determine whether infant sex affected each measurement. The sample size was determined based on the protease activity and inhibitors’ concentrations detected in our previous study (2, 7).

## Results

### Maternal demographics

We collected human milk samples from 20 EP-delivering mothers (GA 24–27 weeks), 12 VP-delivering mothers (GA 28–32 weeks) and eight term-delivering mothers (GA 38–39 weeks) (Table 1). The postnatal age at sampling and maternal age did not significantly differ among the three groups.

### The plasmin system in human milk

The overall average values of the plasmin system in human milk samples from all lactating women are represented in Table 2. Plasmin activity, uPA, and tPA were detected in all human milk samples. Human PAI-1 was detected in 17 out of 40 milk samples (42.5%), whereas the PAI-1/uPA complex was present in 13 out of 40 milk samples (32.5%). The PAI-1/tPA complex was not detected in any milk sample (data not shown).

**TABLE 2:** Measurements for the plasmin system in human milk from all lactating women in the study.

Measurements	Average ± SD (min, max)^a^
Plasmin activity (U/mL)	4.2 ± 2.4 (1.3–11.3)
Human urokinase-type plasminogen activator (uPA) (ng/mL)	0.66 ± 0.43 (0.12–1.9)
Human tissue-type plasminogen activator (tPA) (ng/mL)	4.1 ± 3.7 (0.83–14.2)
Human plasminogen activator inhibitor type 1 (PAI-1) (U/mL)	0.62 ± 1.0 (0.11–5.0)
PAI-1/uPA complex (ng/mL)	0.76 ± 1.9 (0.007–9.4)
PAI-1/tPA complex (ng/mL)	ND

^a^Milk samples from mothers (n = 40) who delivered extremely preterm (n = 20), very preterm (n = 12), and term infants (n = 8). ND, not detected.

### Effect of prematurity on the plasmin system in human milk

**FIGURE 2:**
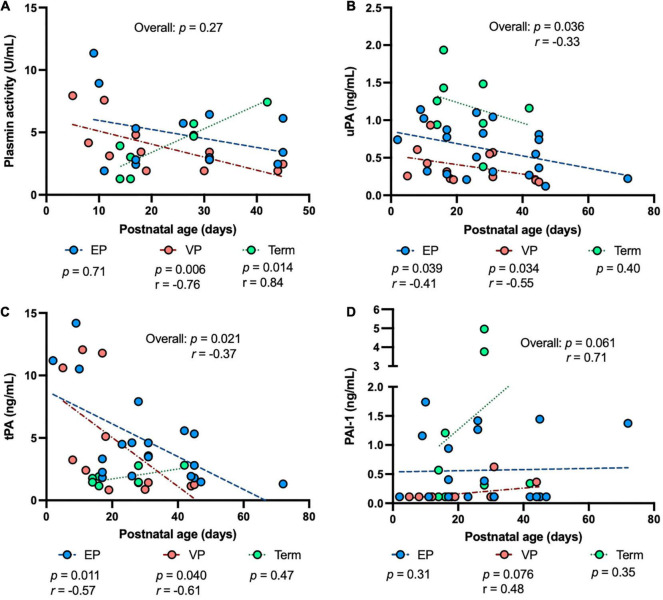
The levels of plasmin system components in milk from extremely preterm-(EP), very preterm-(VP), and term-delivering mothers across postnatal age at sampling. **(A)** Plasmin activity across postnatal age; **(B)** human urokinase-type plasminogen activator (uPA) across postnatal age; **(C)** human tissue-type plasminogen activator (tPA) across postnatal age; **(D)** human plasminogen activator inhibitor type 1 (PAI-1) across postnatal age in human milk from EP (*n* = 20), VP (*n* = 12), term groups (*n* = 8), and combined groups (overall, *n* = 40). *r*, Spearman correlation.

To determine the impact of prematurity on the plasmin system in human milk, we examined the levels of human uPA, tPA, PAI-1, the PAI-1/uPA complex and plasmin activity in human milk from women who delivered EP, VP or term infants across postnatal age at sampling, GA at birth, postmenstrual age (PMA) at sampling. The plasmin activity in human milk was correlated negatively with postnatal age at sampling in the VP groups (*P* = 0.006) and was correlated positively in the term group (*P* = 0.014) but did not correlate in the EP group (Figure 2A). Human uPA (Figure 2B) and tPA (Figure 2C) concentrations in human milk were correlated negatively with postnatal age at sampling in both EP and VP groups and when combined with the three groups, but no correlation was detected in the term group alone. Human PAI-1 concentration tended to be correlated positively with postnatal age in the EP group and when combined with the three groups (Figure 2D). No correlation was detected between the PAI-1/uPA complex concentration and postnatal age (Supplementary Figure 1A).

**FIGURE 3:**
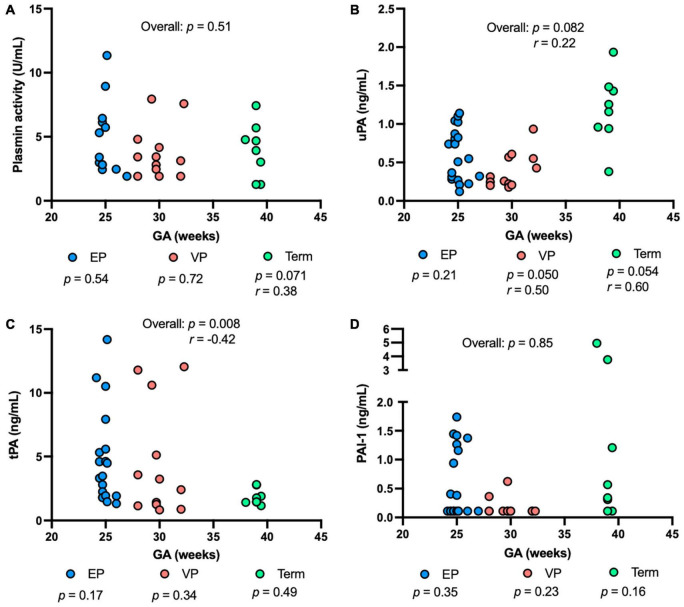
The levels of plasmin system components in milk from extremely preterm-(EP), very preterm-(VP), and term-delivering mothers across gestational age (GA) at birth. **(A)** Plasmin activity across GA; **(B)** human urokinase-type plasminogen activator (uPA) across GA; **(C)** human tissue-type plasminogen activator (tPA) across GA; **(D)** human plasminogen activator inhibitor type 1 (PAI-1) across GA in human milk from EP (*n* = 20), VP (*n* = 12), term groups (*n* = 8), and combined groups (overall, *n* = 40). *r*, Spearman correlation.

Plasmin activity tended to be correlated positively with GA in the term group (*P* = 0.071), but no correlation was detected in either the EP or VP group across GA (Figure 3A). uPA concentration was correlated positively with GA in the VP group (*P* = 0.050, Figure 3B). uPA concentration tended to increase with increasing GA in the term group (*P* = 0.054) and when combined the three groups (*P* = 0.082) but did not change in the EP group (Figure 3B). tPA concentration negatively correlated with GA when combined with the three groups (*P* = 0.008) but no correlation was detected in the individual group (Figure 3C). PAI-1 (Figure 3D) or PAI-1/uPA (Supplementary Figure 1B) concentration was not affected by the GA.

**FIGURE 4:**
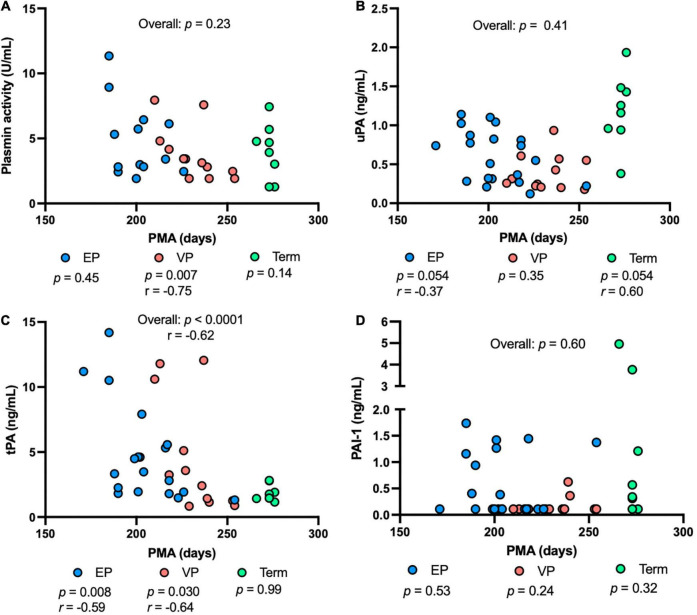
The levels of plasmin system components in milk from extremely preterm-(EP), very preterm-(VP), and term-delivering mothers across postmenstrual age (PMA). **(A)** Plasmin activity across PMA; **(B)** human urokinase-type plasminogen activator (uPA) across PMA; **(C)** human tissue-type plasminogen activator (tPA) across PMA; **(D)** human plasminogen activator inhibitor type 1 (PAI-1) across PMA in human milk from EP (*n* = 20), VP (*n* = 12), term groups (*n* = 8), and combined groups (overall, *n* = 40). *r*, Spearman correlation.

Plasmin activity decreased with increasing PMA in the VP group (*P* = 0.007), but no correlation was detected in the EP group, term group, or combined groups (Figure 4A). uPA concentration tended to decrease with increasing PMA in the EP group and tended to be correlated positively with PMA in the term group (*P* = 0.054) (Figure 4B). tPA concentration was correlated negatively in the EP (*P* = 0.008) and VP (*P* = 0.030) groups but did not correlate in the term group (Figure 4C). PAI-1 (Figure 4D) or PAI-1/uPA (Supplementary Figure 1C) concentration was not affected by the PMA.

The measurements did not change across maternal age. None of the measurements was significantly affected by infant sex.

## Discussion

Plasmin can act on milk proteins within the mammary gland (1) and may continue to enhance the hydrolysis of milk proteins within the infant’s stomach (2). This potential supplement to the infant’s own proteolytic system could be especially important for preterm infants, who have lower gastric digestive capacity than term infants (7). Adequate functioning of protein digestion is critical to sustaining the metabolic needs of the newborn. The breakdown of proteins releases free essential and non-essential amino acids that are used by cells for protein synthesis or energy production. Moreover, neonatal protein digestion releases fragments of proteins—peptides—that may exert bioactivity within the infant’s gut and systemically (10–13).

In this study, we quantified the plasmin system components in human milk, including plasmin activity, the activators (uPA and tPA), plasminogen activator inhibitor (PAI-1), and the PAI-1/uPA complex (Graphical Abstract). Plasmin activities measured [4.2 ± 2.4 (1.3 − 11.3) U/mL] were in the same ranges as those observed Korycha-Dahl et al. (14) for human milk collected 6–7 days postpartum, unknown GA (4.8 ± 4.2 U/mL). Plasmin activity was measured rather than plasmin concentration as we previously reported that plasmin concentration in human milk of preterm-delivering mothers was not influenced by GA or postnatal age (6). Moreover, we decided a substrate assay-based activity measurement would provide a better understanding of how plasmin is affected by the presence of uPA, tPA, and PAI-1 than an ELISA measurement.

The detected concentration of tPA [4.1 ± 3.7 (0.83–14.2) ng/mL] aligns with that determined by Ishii et al. (15) for 24 term-delivering, healthy women providing milk samples at 1–210 days of lactation (2.8–20 ng/mL). We also quantified the content of uPA in human milk [0.66 ± 0.43 ng/mL (0.12–1.9)], which has not been previously reported in the literature. tPA was higher than uPA in human milk. Previous studies indicate that uPA is more associated with the whey component, whereas tPA is mostly associated with the casein micelle, due to differing targeting determinants in the non-catalytic domains of tPA and uPA (9). tPA on the casein micelle may help activate the plasminogen that is also there (4). uPA was present more frequently complexed with PAI-1 than alone, suggesting that “uPA was mostly in a blocked form.” PAI-1/tPA complex was not detected in the human milk samples. This absence of PAI-1/tPA could be because the tPA is mostly bound to the casein micelle, whereas the PAI-1 is mostly in the serum/whey phase, preventing their interaction. No study has previously measured the PAI-1/uPA complex, the PAI-1/tPA complex or PAI-1 in human milk or in bovine milk.

The balance of plasminogen activators (tPA and uPA) and the plasminogen activator inhibitor (PAI-1) might promote a controlled balance of proteolysis of milk proteins, allowing the release of some bioactive milk peptides and free amino acids (16, 17) while preserving some intact bioactive proteins within the mammary gland and the infant digestive tract. Such a balance may allow for the protein component of milk to exert effects throughout the mammary gland and the human infant as bioactive proteins and peptides while providing amino acid nourishment for the infant. Whether the plasmin system present in human milk plays a specific physiological role in milk remains unclear.

We assessed the differences in plasmin activity and in concentrations of tPA, uPA, PAI-1, and the PAI-1/uPA complex in human milk among the EP-, VP- and term-delivering groups. Our results showed that uPA concentration was higher in early postnatal age in premature-delivering mothers (EP and VP) than in late postnatal age. However, uPA concentration tended to be reduced in early premature GA compared to term GA in human milk. A previous study demonstrated that milk protein concentrations decreased with increasing postnatal age in preterm milk samples during the first 2 months after birth, but protein concentration was higher in preterm milk than in term milk (18). uPA is produced by blood monocytes and milk macrophage cells (17, 19). The serine protease uPA plays a role in pericellular proteolysis during cell development and tissue remodeling following tissue injury and inflammatory responses (20, 21). Whether the amount of uPA ingested in preterm infants fed their parent’s milk influences their innate immunity and inflammatory responses remains to be investigated. The role of uPA in infant digestion has not been examined.

We observed that human tPA concentration in human milk decreased with increasing postnatal age, GA, and PMA in the EP- and VP-delivering mothers, suggesting that tPA is strongly influenced by prematurity and postnatal age. Likewise, tPA concentration in human milk decreased gradually with increasing postpartum time (from day 1 to 210) in 24 term-delivering healthy women (15). Similarly, α_1_-antitrypsin [which is a plasmin inhibitor (4)] and anti-chymotrypsin in human milk (protease inhibitors against trypsin, chymotrypsin and elastase) decreased with increasing postpartum time during the first week of life (22). The serine protease tPA is secreted from endothelial cells in blood vessels to activate plasminogen into plasmin and catalyzes fibrin (15). tPA may play a role in restructuring breast tissue and human milk secretion as tPA enhances the canalization of mammary ducts in lactation (17). The role of tPA in infant digestion has not been examined.

Plasmin activity in human milk decreased with increasing postnatal age and PMA in the VP group but was correlated positively with postnatal age in the term group and not influenced in the EP group, indicating that the plasmin activity across postnatal age was affected by the degree of prematurity at birth. Our previous study found that active plasmin concentration in human milk from preterm-delivering mothers did not change across GA but tended to decrease with increasing postnatal age (6). This difference in the effect of postnatal age between the two studies could be due to methodological differences, as the previous study measured plasmin *via* ELISA, whereas this present study used a fluorometric activity assay. Armaforte et al. observed that plasmin activity was higher in milk from premature-delivering mothers (30–37 weeks of GA, unspecified postnatal time) than that from term-delivering mothers (born after 38 weeks of GA, within 4 weeks after the colostral period) (23). The differing results between Armaforte et al.’s work and our own may result from differing characteristics of the preterm-delivering mothers, including GA and lactation time. A limitation of our study is the small sample size, which could explain the lack of difference in plasmin activity observed across GA.

Plasmin activity, uPA and tPA were detected in all human milk samples. Human PAI-1 was detected in 17 out of 40 milk samples (42.5%), whereas the PAI-1/uPA complex was present in 13 out of 40 milk samples (32.5%). The PAI-1/tPA complex was not detected in any milk sample.

We observed that PAI-1 and PA1/uPA complex concentrations remained stable across GA, postnatal age and PMA. Under normal conditions, PAI-1 is present in plasma at low concentrations (5–20 ng/mL). The most abundant pool of PAI-1 is present in platelet granules, containing 90% of the circulating PAI-1 (23). PAI-1 present in human milk could derive from paracellular transfer across the blood-milk barrier (24). Åstedt et al. (25) reported that PAI-1 activity in the umbilical cord was higher in term newborns than in preterm infants.

In summary, active plasmin, uPA and tPA were present in all milk samples, PAI-1 and the PAI-1/uPA complex were present in some samples and the PAI-1/tPA complex was not present in any samples. Premature delivery impacted the plasmin activity and the concentrations of two plasminogen activators (uPA and tPA) and PAI-1 in human milk. Whether these changes in plasminogen activators and the plasminogen activator inhibitor in milk across gestational age at delivery impact the balance of proteolytic digestion within their respective infants (which could impact growth and immune development) remains to be investigated. This difference may be of particular importance for preterm infants who receive donor human milk which is pasteurized and generally comes from mothers who delivered at term and have well-established lactation (increased postnatal age). These factors can affect inhibitors and activators present in human milk.

## Data availability statement

The raw data supporting the conclusions of this article will be made available by the authors, without undue reservation.

## Ethics statement

The studies involving human participants were reviewed and approved by University of California, Davis and Oregon State University. The patients/participants provided their written informed consent to participate in this study.

## Author contributions

VD-M designed and conducted the research, analyzed the data, performed the statistical analysis, and wrote the manuscript. MU provided the milk samples. DD provided essential materials and data interpretation and edited the manuscript. VD-M and DD had primary responsibility for the final content. All authors have read and approved the final manuscript.
