# Supplementary material for: Premature delivery impacts the concentration of plasminogen activators and a plasminogen activator inhibitor and the plasmin activity in human milk
Source: Front Pediatr. 2022 Aug 9;10:917179. doi: 10.3389/fped.2022.917179 (PMC9396237; doi:10.3389/fped.2022.917179)
Supplement: Supplementary file 1 [file Data_Sheet_1.pdf]

Supplementary Materials

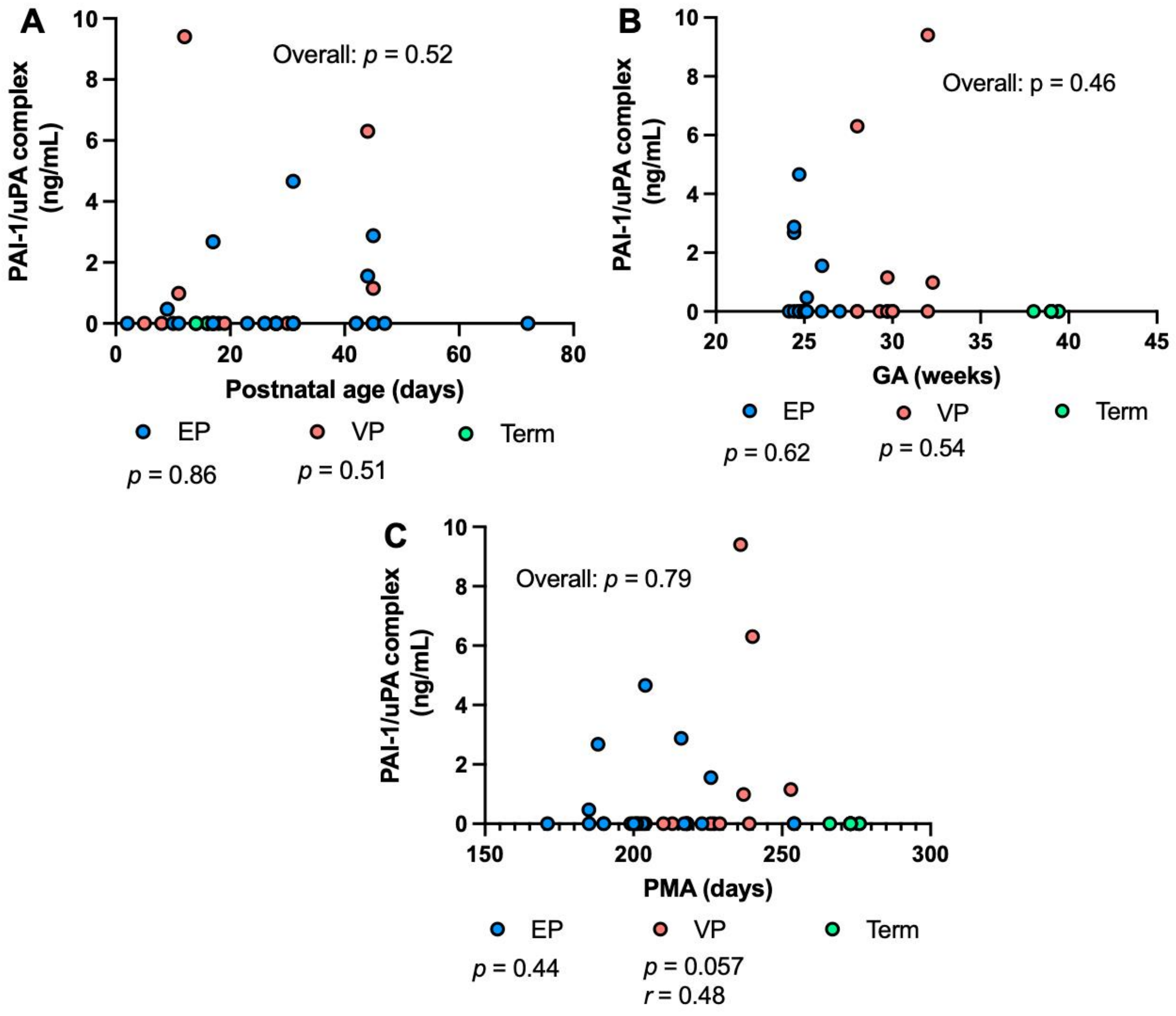

Supplementary Figure 1. The concentration of complex human plasminogen activator inhibitor type 1 and urokinase-type plasminogen activator (PAI-1/uPA) in milk from extremely preterm-(EP), very preterm-(VP) and term-delivering mothers. PAI-1/uPA across postnatal age at sampling, gestational age (GA) at birth, and postmenstrual age (PMA) in human milk from EP (n = 20), VP (n = 12), term groups (n = 8), and combined groups (overall, n = 40). r, Spearman correlation.
